# Ambiguity aversion: bibliometric analysis and literature review of the last 60 years

**DOI:** 10.1007/s11301-021-00250-9

**Published:** 2021-12-13

**Authors:** Christoph Bühren, Fabian Meier, Marco Pleßner

**Affiliations:** 1 Hamm-Lippstadt University of Applied Science: Hochschule Hamm-Lippstadt, Hamm, Germany; 2 Department of Economics, University of Kassel, Kassel, Germany

**Keywords:** Ambiguity aversion, Bibliometric analysis, Literature review, Ellsberg paradox

## Abstract

We conduct a bibliometric analysis and review the literature of the last six decades on ambiguity aversion. Comparing trends in theoretical, experimental, and empirical contributions, our study presents the main aspects that are discussed in this literature. We show the increasing relevance of ambiguity aversion for decision-making research and discuss factors influencing attitudes on ambiguity. Our literature review reveals unsolved problems in the research on ambiguity and gives an outlook on new ventures for future research.

## Introduction

Daily, we decide under uncertainty. In some of these decisions, we know the objective probabilities of the underlying alternatives (decisions under risk) or can at least assess subjective probabilities (decisions under uncertainty in the narrow sense) (Knight 1921). But sometimes, we have to decide under ambiguity – without having a clue on the probabilities of the outcomes. Typically, people feel uncomfortable deciding under ambiguity and try to avoid these decisions, they are ambiguity-averse. This can lead to systematic biases (Ellsberg 1961) – especially to the violation of the independence axiom, which demands that rational decisions should be independent of outcomes that all alternatives have in common.

The research field of ambiguity aversion has experienced a boom of publications in the past two decades. It all began with the seminal paper of Ellsberg (1961), followed by both theoretical and experimental contributions. Recent theoretical applications of the decision-theoretic models are, for instance, Gao and Driouchi (2018), Bergen et al. (2018), and Dicks and Fulghieri (2019). Ryall and Sampson (2017) and Anderson (2019) are examples of recent empirical studies on ambiguity aversion. Furthermore, the corona pandemic yields several examples for decisions under ambiguity (Durodié 2020; Gassman et al. 2021; Kishishita et al. 2021). The probabilities of the effects of anti-corona measures are very hard to assess. Thus, decisions on these measures are likely to be prone to biases. Also, several exogenous factors influence decisions in pandemic management that are even harder to asses, such as time pressure on decision-makers or ambiguity about personal vaccination decisions (Courbage and Peter 2021; Lipscy 2020).

Since 1961, ambiguity aversion has been inspiring several strands of theoretical and experimental literature in decision theory, economics, psychology, and behavioral economics. The goals of our paper are (1) to show the development of this literature (Sect. 2.1), (2) to present an overview of its essential findings (Sects. 3 and 4), and (3) to identify research gaps and room for future research (Sects. 5 and 6.1).

Literature reviews on ambiguity aversion have already been published. Camerer and Weber (1992) review the early literature on ambiguity aversion, while Trautmann and van de Kuilen (2015) and Al-Najjar and Weinstein (2009) discuss more recent contributions. Etner et al. (2012) review the literature on ambiguity aversion in decision theory and Guidolin and Rinaldi (2013) in asset pricing. Our contribution is to provide an overall overview of both theoretical and empirical papers. Furthermore, we cover the entire period of 60 years of research on ambiguity aversion. Finally, we are the first to publish a bibliometric analysis on ambiguity aversion. Recent bibliometric studies on other streams of literature, for instance, Keding (2021) and Ozturk (2021) in the field of strategic management, show the fruitfulness of this analysis.

We follow Block and Fisch (2020) in conducting a reproducible, standardized literature search with a specified research goal and providing a map of the research field. Our study starts with the bibliometric analysis on the development of research on ambiguity aversion based on 556 publications. In line with Pleßner (2017), we extract the documents from the search platform EBSCOhost. We present the development of the number of publications and authors over time and analyze the ratings of these publications. Moreover, we distinguish six clusters of publications (e.g., experimental vs. theoretical) and study the co-occurrence of keywords. The analysis highlights the most important papers and authors in the field.

Our data set used for the bibliometric analysis (Sect. 2) also serves as the basis for our literature overview (Sects. 3–5). In this review, however, we focus on 91 papers from the bibliometric analysis that are ranked as A and B in JOURQUAL3. To reduce the probability that we miss relevant papers from journals without ranking, from journals ranked worse than B, or from discussion paper series, we manually add further 40 titles. We separate these 131 publications into theoretical and experimental/empirical contributions. First, we describe the *Subjective Expected Utility* (SEU), *Choquet Expected Utility* (CEU), *Maxmin Expected Utility* (MEU), and *Smooth Ambiguity Model* (SAM) as well as theoretical applications of these models. Distinct to Machina and Siniscalchi (2013), we limit our review to the four most cited theoretical models in the Business Source Premier database in EBSCOhost (see Appendix F for an overview). Second, we consider the evidence for ambiguity aversion concerning (1) laboratory experiments testing the models, (2) gains and losses with varying probabilities of the risky alternative, (3) ambiguity premiums, and (4) experimental and empirical applications.

## Bibliometric analysis

**Fig. 1:**
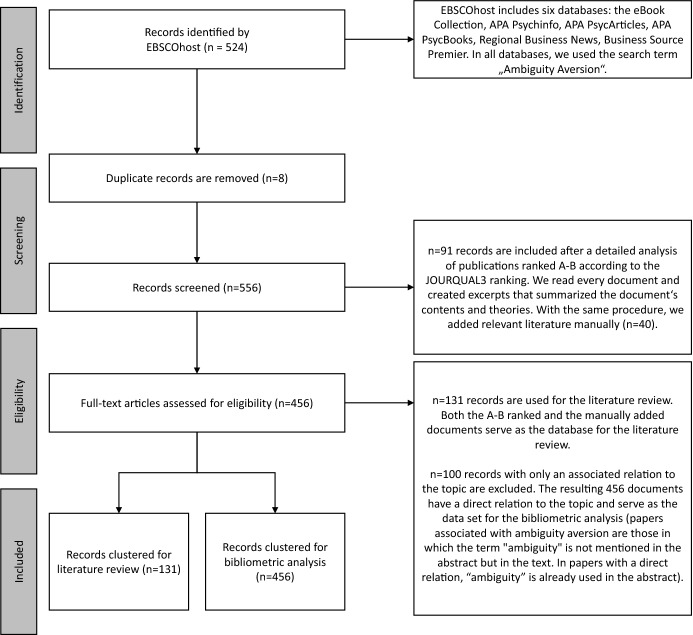
Methodological steps of our bibliometric analysis and our literature review

Figure 1 describes the procedure of our literature review and bibliometric analysis. We follow Shaffril et al. (2019) and Det Udomsap and Hallinger (2020) in applying the PRISMA standard for our literature search. As the *resource,* we use 524 publications in the bibliometric analysis. For our analysis, we mainly focus on the title, abstract, and year of publication. We conduct the literature search on the EBSCOhost platform using six databases (the eBook Collection, APA Psychinfo, APA PsycArticles, APA PsycBooks, Regional Business News, and Business Source Premier) with the search term “ambiguity aversion''. EBSCOhost has also been used by Pleßner (2017) for a bibliometric analysis on the disposition effect. We apply the same search key string for all six databases. Most of the 524 found documents are journal articles (471), others are monographs (24), dissertations (17), and articles in anthologies (12).

In the *screening* stage, we had to remove 8 duplicates from the data basis (e.g., working papers that became journal articles). This results in 516 documents. In the literature review, we mainly focus on sources published in journals with a high ranking (91 titles published in journals with the ratings A + to B according to the JOURQUAL3 ranking). On the one hand, our sample is, thus, likely to be of high quality and impact. On the other hand, the focus on these journals can lead to a retrieval bias in the selection of the literature (Cooper et al. 2018). We try to reduce this bias in the *screening* stage by manually adding relevant literature that are not necessarily part of the JOURQUAL ranking. As the search for “ambiguity aversion'' does not automatically find all relevant papers, we added 40 additional documents from the literature review in Sects. 3 and 4. We identified the additional documents by reading all 91 documents carefully. Afterward, we created excerpts that summarized the papers' main contents. We integrated documents that were mentioned frequently and that were not already part of our data set. This results in 131 documents that are used for the literature review. Nevertheless, the possibility that some publications are still missing cannot be ruled out. After the *screening* stage, we end up with 556 documents (524 minus 8 duplicates plus 40 manually added documents).

These 556 articles enter the *eligibility* stage. Here, we divide the papers into a group with direct relation (82%) and a group with associated relation to ambiguity aversion (18%). Papers associated with ambiguity aversion are those in which the term “ambiguity'' is not mentioned in the abstract but in the text. These documents are excluded from further analysis. In papers with a direct relation, “ambiguity” is already used in the abstract. Finally, we use 456 documents for the bibliometric analysis and 131 documents (91 + 40) for the literature review.

Based on a content analysis, we cluster the publications into “decision-theoretical model'', “experimental study'', “empirical study”, “survey”, “model application (without data)” and “comment”. The cluster *decision-theoretical model* includes all publications that either develop a new model or further develop an existing model. An empirical paper that is based on quantitative analyses is labeled *empirical study*. A publication containing an experiment is categorized as *an experimental study*. The category *model application without data* encompasses publications that either apply a model to a theoretical problem or include theoretical analyses without any experimental or empirical evidence. The *comment* cluster only contains comments without own data or theory.

**Fig. 2:**
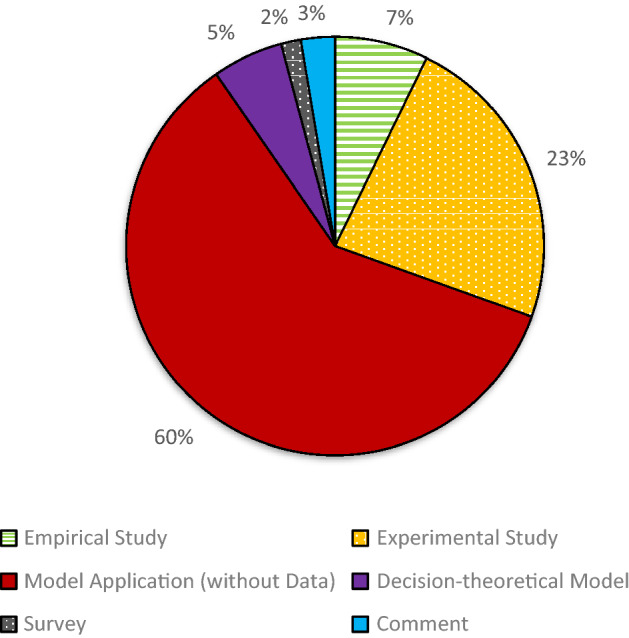
“Direct Relation” publications by subcategory

Figure 2 shows that the majority of publications with direct relation to ambiguity aversion can be assigned to the clusters *model application (without data), experimental study, and empirical study.* 5% of these publications belong to the category *decision-theoretical model*. Especially the publications of the cluster *model application (without data)* are connected to the cluster *decision-theoretical model*. These applications are typically based on such a model. As an example, Altug et al. (2020) analyze the cyclical dynamics of a real business cycle model with ambiguity-averse consumers using the model of Klibanoff et al. (2005) (see Sect. 3.4).

### Development over time

**Fig. 3:**
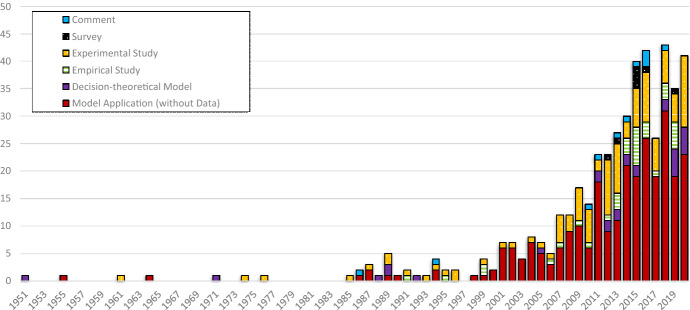
Number of publications with “Direct Relation” over time

The number of publications on ambiguity remained relatively low until the beginning of the 2000s (see Fig. 3). After that, it rose sharply until the end of our observation period. The highest number of annual publications is recorded in 2018 with 43. In the years 1951–1999, an average of 0.76 papers are published. In the years 2000–2020, it is 20.2. This dynamic is in line with the development of behavioral economics analyzed by Costa et al. (2019) showing an exponential increase of publications between 2000 and 2015. Using the search terms “behavioral economics, “behavioral finance”, and “behavioral accounting”, Costa et al. (2019) observe 1–3 publications per year between 1967 and 1990, less than 30 publications per year between 1991 and 2001, over 100 in 2008, 250 in 2012, and 346 publications in 2015. Similarly, in the behavioral finance literature, Pleßner (2017) identifies two papers on the disposition effect in 2000 but 26 in 2014 and Jain et al. (2021) merely count one paper on behavioral biases in 1995 but 28 in 2019. In our data, the increasing trend of publications between 2000 and 2015 continues in the years 2016 and 2018. However, in 2017, the number of publications on ambiguity aversion decreases compared to the previous year. This could be interpreted as the beginning of a period of stagnation in the number of publications. The lower publication numbers in 2019 and 2020 compared to 2018 support this interpretation. Figure 3 also shows the distribution of our five clusters over time. In nearly every year, most of the papers are *model applications* (Gao and Driouchi 2018; Lo 1998; Turocy 2008). This does not correspond to the review by Goyal and Kumar (2021), who observe 86% empirical studies, 10% conceptual studies, 3% reviews, and 1% meta-analyses on financial literacy from 2000 to 2019. But also in the field of ambiguity aversion, *empirical* and *experimental studies* have been gaining ground from the 2000s onwards (Dimmock et al. 2016b, 2016a; Koudstaal et al. 2016; Muthukrishnan et al. 2009; Sutter et al. 2013). The low proportion of qualitative studies is striking. The proportion of comments is also low.

Table 1 analyzes the average number of authors per publication. In the early development of the research field – from 1961 until 1990 – more than 40% of the papers were single-authored (1.71 authors on average), in the recent development – from 2011 until 2020 – around 20% (2.35 authors on average). This is in line with the development of co-authorship in the literature of economics in general. Analyzing the RePEc archive, e.g., Rath and Wohlrabe (2016) observe an increase from 1.56 authors per paper in 1991 to 2.23 authors in 2013. This reflects the general trend and increased importance of intra- and interdisciplinary collaboration in behavioral economics among theorists, experimentalists, and empirics.

**Table 1:** Average number of authors by period

Period	Average number of authors	Share (%) of single-author publications
2011–2020	2.35	20.43
2001–2010	2.01	31.46
1991–2000	1.90	25.00
1961–1990	1.71	41.18

In addition to the number of publications, we examine the journal rankings of the papers based on JOURQUAL3 (Henning-Thurau et al. 2004). The ranking assesses the quality of the journals from the best category “A + ” to the worst “D”. The overall ranking includes several sub-rankings, e.g., “General Business Studies” or “Banking/Finance”. It should be noted that a large number of publications (321 of 456) are not part of the ranking. They are either listed in other rankings (e.g., SJR), or they do not have any ranking. These 321 publications are published in 125 different journals and 10 discussion paper series. The five journals in which these papers are published most frequently (*Journal of Risk and Uncertainty, Journal of Economic Theory, Journal of Economic Behavior and Organization, Journal of Mathematical Economics*; 21.8 papers on average per journal) have an average SJR ranking of 0.129. The 20 journals with the most frequent publications on ambiguity aversion outside JOURQUAL (8.4 papers on average per journal) have an average ranking of 0.148.

**Fig. 4:**
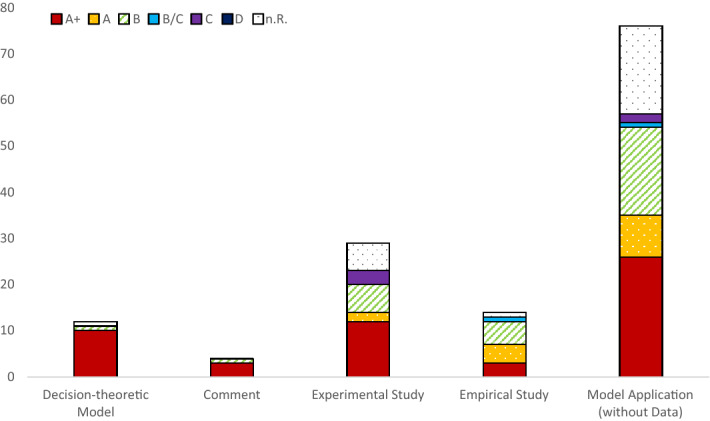
JOURQUAL3-rankings by cluster

Most of the 135 papers analyzed in Fig. 4 are published in journals ranked “A + ” or “A” in JOURQUAL. For *decision-theoretical models* and *comments*, the proportion of publications rated “C” or worse is zero. 74.8% of the *experimental studies, empirical studies*, and *model applications (without data)* are published in journals rated “A + ”, “A”, or “B”. The share of *model applications* published in A + journals (34.2%) is lower than that of *experimental studies* (41.4%).

According to Google Scholar,^1^ the clusters also differ in their citation frequency: The average number of citations in the clusters *decision-theoretic model* (113.5) and *experimental study* (100.0) is much higher than in *model applications (without data)* (59.5) and *comments* (8.1). *Empirical studies* are cited 82.5 times on average. Web of Science also reports the highest citation numbers for *decision-theoretic models* (47.1) and *experimental studies* (43.6), followed by *empirical studies* (26.5), *model applications (without data)* (22.2), and *comments* (2.4). The Business Source Premier database (via EBSCOhost) indicates a lower number of citations per cluster. According to this database, the citation frequencies of *decision-theoretic models* (15.3) and *experimental studies* (11.2) are higher than that of *model applications (without data)* (5.3) and *comments* (0.7). *Empirical studies* are cited nearly as much as *experimental studies*, 10.4 times on average.

**Fig. 5:**
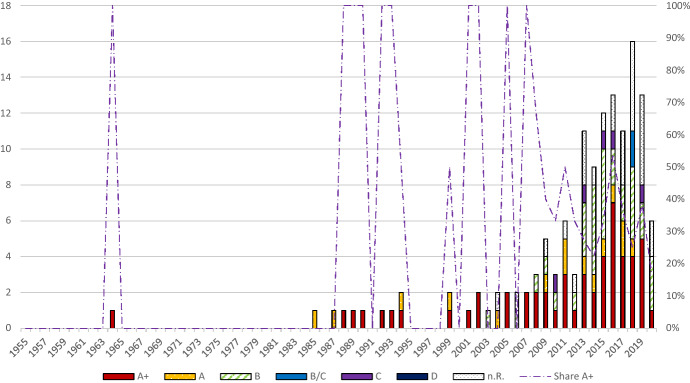
JOURQUAL3-rankings over time

Figure 5 shows the JOURQUAL journal rankings of papers on ambiguity aversion over time. The absolute scale of the ordinal axis refers to the number of publications in the respective year, while the relative scale shows the proportion of A + publications. Considering the whole period – including years in which there are no publications in ranked journals – the average proportion of A + papers is 23.9%. Excluding years with no ranked publications, the average share of A + journals is 52.6%. Thus, ambiguity aversion is very relevant and of general interest. The share of top publications decreases after 2006 because of the increasing number of publications on ambiguity aversion. The absolute number of A + publications is highest in 2017 with seven papers.

Citation frequencies in Google Scholar also differ between journal rankings: While publications in A + -ranked journals are cited 229.9 times on average, papers are cited 135.2 times on average if they are published in an A-ranked journal. In B-ranked journals, they are cited 17.2 times, and in C-ranked ones 6.5 times. Not-ranked journals have a citation rate of 35.1 on average. Web of Science reports lower levels of citation frequencies: A + papers are cited 85.9 times, A papers 53.6 times, B publications 6.8 times, and C publications 2.8 times on average. Not-ranked journals have an average citation frequency of 15.5 in Web of Science. Using the Business Source Premier database, EBSCOhost finds 26.9 citations for papers in journals ranked A + and 16.4 in those ranked A. B-ranked articles are cited 1.5 times and C-ranked ones 1.2 times. Papers that are not ranked in JOURQUAL are cited 3.9 times on average according to EBSCOhost.

### Important papers, authors, and keywords

Table 2 shows that the most prominent publication in the field of ambiguity aversion is the paper that started this line of research. According to Google Scholar, Ellsberg (1961) has already been cited by 9411 papers, EBSCOhost finds 831 citations from the Business Source Premier database. Moreover, the theoretical models of Gilboa and Schmeidler (1989), Schmeidler (1989), and Klibanoff et al. (2005) (see Sects. 3.2–3.4) are cited very frequently. Heath and Tversky (1991), Judge et al. (1999), and Chen and Epstein (2002) are connected to these models. Table 2 and Fig. 3 reveal that theoretical papers set the basis of the research field of ambiguity aversion and are most cited up to now. Since 2000, a lot of experiments and empirical papers have been testing these models (Eichberger et al. 2012; Halevy 2007; Hey et al. 2010, see also Sect. 4). At least by now, however, these publications have been cited less often. Table 3 complements this finding by showing the five most-cited authors in the field. Besides the top-cited author Ellsberg, some prominent theorists co-author many theoretical applications or experimental tests of their models.

**Table 2:** Important publications in the field

Source	Google scholar citations	Web of science citations	Business source premier citations
Ellsberg (1961)	9411	3210	885
Gilboa and Schmeidler (1989)	5036	1854	732
Schmeidler (1989)	3776	1490	502
Heath and Tversky (1991)	1979	657	253
Klibanoff et al (2005)	1860	687	280
Savage (1951)	1560	NA	NA
Judge et al. (1999)	1524	474	196
Hansen and Sargent (2001)	1214	NA	168
Chen and Epstein (2002)	1128	433	2
Ghirardato et al. (2004)	992	390	173
Abdellaoui et al. (2011)	553	193	63
Sutter et al. (2013)	530	215	88

Citation counts were retrieved on October 2, 2021

**Table 3:** Top-7 authors in the field

Author	Google Scholar Citations	Web of Science Citations	Business Source Premier Citations
Ellsberg, Daniel	9413	3226	831
Schmeidler, David	8812	3344	491
Gilboa, Itzhak	5379	1854	707
Marinacci, Massimo	4509	1453	306
Epstein, Larry G	3812	1754	153
Tversky, Amos	2746	657	249
Klibanoff, Peter	2209	696	278

Citation counts from Google Scholar and Business Source Premier were retrieved on May 3, 2021. Citation Counts from Web of Science were retrieved on October 2, 2021

**Fig. 6:**
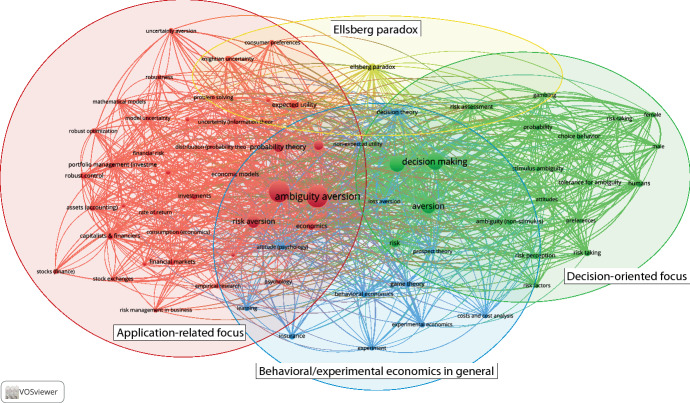
Co-occurrence of keywords (Created using VOS Viewer)

Furthermore, we conduct a co-occurrence analysis of the papers’ keywords. Figure 6 presents the mapping of the most frequently used keywords. We set the minimum threshold for an appearance on the map at 12. This criterion is met by 77 keywords. The size of the nodes in the figure indicates the frequency of the keywords. We see at least three topic areas. The left one shows an application-related focus on financial risk management, portfolio decision problems, and investments in general. Especially the literature we discuss in Sects. 3.5 and 4.4 is represented in this field, e.g., Anderson (2019; keyword: *economic models*), Vardas and Xepapadeas (2015; keyword: *expected* utility), or Dicks and Fulghieri (2019; keyword: *risk aversion*). The area on the right shows a decision-oriented focus (ambiguity tolerance, choice behavior, risk-taking). The literature located in this topic area can be found in particular in Sects. 3.2, 3.3, and 3.4 (e.g., Trojani and Vanini 2004 with the keyword *decision making*). The category in the middle bottom of Fig. 6 represents behavioral and experimental economics in general (e.g., Peysakhovich and Naecker 2017 use the keyword *behavioral economics*, see Sect. 6.1). The keyword *Ellsberg paradox* (in the middle on top) combines these three categories. It is for example used by Klibanoff et al. (2005), who present a new decision-theoretic model of ambiguity aversion. The most used keywords and the keywords with the most connections to others are (of course) ambiguity aversion, decision making, uncertainty, aversion, risk aversion, and probability theory. We can see from the interconnection of the categories that the keywords are centered around decision theory (building or applying models to describe human decision processes under uncertainty and ambiguity theoretically) and behavioral applications (theoretical or experimental and empirical analyses of how ambiguity aversion affects economic decision making). In Appendix A–E, we split the data set into five periods showing the development of the keywords’ co-occurrence. This analysis indicates a trend of the research field from theoretical contributions to experimental and empirical applications.

### Interpretation and discussion of bibliometric results

Our bibliometric analysis shows a significant rise in the number of publications during the last six decades, especially a boom of papers from the 2010s onwards. Simultaneously, the average number of authors per publication rises, which speaks for the increasing importance of collaboration. The development of the research field is in line with the results of other bibliometric research in economics and especially behavioral economics (Rath and Wohlrabe 2016; Pleßner 2017; Costa et al. 2019; Jain et al. 2021). The topics of the publications in the field of ambiguity aversion cluster around decision theory (Schmeidler 1989; Klibanoff et al. 2005; Etner et al. 2012) and behavioral applications (Dimmock et al. 2016a; Stahl 2014; Ellsberg 1961). Most of the papers are either theoretical model applications or empirical and experimental studies that test the theoretical propositions. Few publications are dedicated to the development of theoretical models. We observe a high but decreasing proportion of A + and A publications and an increase of publications with a lower ranking. Contributions on new theoretical models have the highest share of A + publications. Ellsberg’s (1961) seminal paper on his paradox and further behavioral theories build the basis of research on ambiguity aversion and have been cited most up to now, followed by model applications (Gao and Driouchi 2018), experimental papers (Stahl 2014), and empirical research (Anderson 2019). In recent years, the majority of papers have been data-driven (Altug et al. 2020; Anderson 2019; Brenner and Izhakian 2018).

Researchers may find our bibliometric analysis useful to get an overview of the important developments and trends in this stream of literature and to discover the potential for future research publishable in high-quality journals. Nevertheless, our approach of extracting and screening the papers for our literature review (see Fig. 1) might lead to biases, as we had to manually add literature to the bibliometric analysis based on excerpts. In the next two sections, we review the basic theories on ambiguity aversion as well as their theoretical applications (Sect. 3) and present experimental and empirical evidence based on these models (Sect. 4).

## Theory

**Fig. 7:**
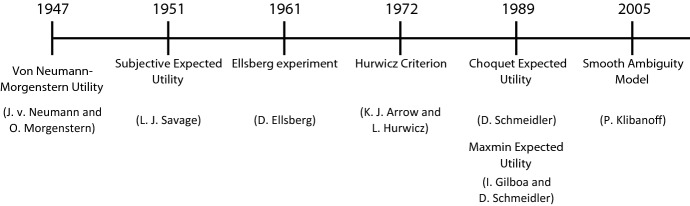
Timeline of theoretical models linked to ambiguity aversion

Figure 7 summarizes the timeline of theoretical models connected to the Ellsberg (1961) paradox. The Ellsberg experiment is not the beginning but seemingly the turning point in the development of theoretical research on ambiguity (see also Tables 2 and 3). We limit our review to the most cited theoretical models on ambiguity aversion in the Business Source Premier database (see Appendix F). Our review differs from Machina and Siniscalchi (2013) as we combine more recent literature (Agliardi et al. 2016; Gilboa and Marinacci 2016; Zheng et al. 2015) with the most relevant theoretical approaches from the previous literature.

### Subjective expected utility and ambiguity

In Ellsberg‘s (1961) seminal paper, he questiones the *Sure Thing Principle* of Savage‘s (1951) Subjective Expected Utility (SEU) theory with a thought experiment. SEU assumes individuals to assess subjective probabilities to alternatives of decision problems under uncertainty if these cannot be objectively quantified (see the *Expected Utility* theory of von Neumann and Morgenstern 1944). Ellsberg (1961) presents two experimental designs. The first design comprises two urns with 100 balls. Urn A contains 50 white and 50 black balls, urn B has an unknown distribution of white and black balls. An ambiguity-averse decision-maker prefers the known probability in urn A. The preference for urn A, no matter if the decision-maker is paid when (1) a black or (2) a white ball is drawn, leads to a contradiction. The first decision indicates the belief that urn B contains fewer black balls than urn A, the second decision implies the belief that urn B contains more black balls than urn A.

The second experimental design is based on an urn with 90 balls – 30 red balls and 60 blue or yellow balls. In the first decision, the decision-maker receives a payoff if the color is drawn on which he or she betted (either red or blue), in the second decision the decision-maker additionally receives a payoff if a yellow ball is drawn. Table 4 provides an example.

**Table 4:** Numerical example for the three-color experimental design in Ellsberg (1961)

	30	60	
	Red	Blue	Yellow
*f*	100	0	0
*g*	0	100	0
*f*’	100	0	100
*g*’	0	100	100

An ambiguity-averse decision-maker prefers action $$f$$ over $$g$$ (thus he or she would win $100 if a red ball is drawn) and action $$g^{\prime}$$ over $$f^{\prime}$$ (thus winning $100 if either a blue or a yellow ball is drawn). Again, this leads to the contradiction that the decision-maker seems to believe that the urn contains less than 30 blue balls when deciding for $$f$$ but more than 30 blue balls when deciding for $$g^{\prime}$$:

*f* ≿ *g* implies:

$$p_{R} u\left( {100} \right) + 1 - \left[ {p_{R} u\left( 0 \right)} \right] > p_{B} u\left( {100} \right) + 1 - \left[ {p_{B} u\left( 0 \right)} \right] \Leftrightarrow p_{R} > p_{B}$$

*f'* ≿ *g'* implies:

$$\begin{aligned} & \left( {p_{B} \vee p_{G} } \right) u\left( {100} \right) + 1 - \left( {p_{B} \vee p_{G} } \right) u\left( 0 \right) > (p_{R} \vee p_{G} ) u\left( {100} \right) \\ & \quad + 1 - (p_{R} \vee p_{G} ) u\left( 0 \right) \Leftrightarrow p_{B} \vee p_{G} > p_{R} \vee p_{G} \Leftrightarrow p_{B} > p_{R} \\ \end{aligned}$$

After Ellsberg (1961) introduced the experiment, many applications and extensions of the design were published (Di Mauro and Maffioletti 2004; Du and Budescu 2005; Halevy 2007; Moore and Eckel 2006; Yates and Zukowski 1976, see also Sect. 4).

### Choquet expected utility

The Choquet Expected Utility (CEU) model developed by Schmeidler (1989) is based on capacity $v$, which reflects the degree of uncertainty experienced by the decision-maker and his or her attitude towards uncertainty (Agliardi et al. 2016). The capacity represents a set of non-additive probability distributions, and the utility is calculated with the Choquet integral (Etner et al. 2012). Schmeidler (1989) describes the decision criterion as follows:

$$\begin{gathered} f{ \succsim }g\, {\text{is equivalent to}} \hfill \\ \int\nolimits_{Ch} {u\left( f \right)dv} \ge \int\nolimits_{Ch} {u\left( g \right)dv} \hfill \\ \end{gathered}$$

If $S = \left\{ {s_{1} ,s_{2} , \ldots ,s_{n} } \right\}$ and $f\left( {s_{i} } \right) = x_{i} ,i = 1, \ldots ,n$ with $x_{i} \le x_{i + 1}$,

then

$$\begin{aligned} \mathop \int \nolimits_{Ch}^{{}} u\left( f \right)dv = & \, u\left( {x_{1} } \right) + \left( {u\left( {x_{2} } \right) - u\left( {x_{1} } \right)} \right)v\left( {\left\{ {s_{2} ,s_{3} , \ldots ,s_{n} } \right\}} \right) + \cdots \\ & \quad + \left( {u\left( {x_{i} + 1} \right) - u\left( {x_{i} } \right)} \right)v\left( {\left\{ {s_{i + 1} , \ldots ,s_{n} } \right\}} \right) + \cdots + \left( {u\left( {x_{n} } \right) - u\left( {x_{n - 1} } \right)} \right)v\left( {\left\{ {s_{n} } \right\}} \right). \\ \end{aligned}$$

Thus, a decision-maker first considers the worst outcome of an alternative and continues with better ones until the whole outcome space is evaluated. In contrast to Savage's SEU, different utility functions can be applied for different actions (Agliardi et al. 2016). Furthermore, the non-additivity of CEU can be used to model ambiguity aversion. An individual is ambiguity-averse if his or her capacity $v$ is convex and the utility function concave or linear (Schmeidler 1989).

### Maxmin expected utility

While Savage's SEU assumes that an individual prefers a single alternative, the Maxmin Expected Utility (MEU) model assumes that several alternatives can be preferred simultaneously. In the model, the decision-maker chooses the option that promises the maximum of the minimal expected utilities (Gilboa and Schmeidler 1989). Formally, an action $f$ is preferred to an action $g$ if and only if

$$ \mathop{\min }\limits_{p \in C}^{} E_{p} u\left( f \right) \ge \mathop {\min }\limits_{p \in C}^{} E_{p} u\left( g \right)$$

(Etner et al. 2012).

Hence, MEU can model ambiguity aversion. An action $f$ in a state $s$ leads to a prize $x$ with a probability distribution of $f\left( s \right)$. Imagine an experiment in which the decision-maker is allowed to choose between the draw from urn A or B (containing black and white balls each), urn B representing ambiguity. Formally, the process can be described by $\alpha f + \left( {1 - \alpha } \right)g$ with $\alpha \in \left[ {0,1} \right]$. If state $s$ occurs, then the prize the decision-maker wins (outcome) depends on the lottery $\alpha f\left( s \right) + \left( {1 - \alpha } \right)g\left( s \right)$ (Segal 1990). $\alpha$ represents the probability of choosing action $f$, $1 - \alpha$ the probability of choosing action $g$, and $f\left( s \right)$ the probability distribution of the lottery. According to MEU, $\alpha f\left( s \right) + \left( {1 - \alpha } \right)g\left( s \right){ \succsim }f$ applies (Gilboa and Schmeidler 1989). As an example,$f$ can denote the bet on a white ball in urn B, while $0.5f\left( s \right) + 0.5g\left( s \right)$ represents the bet on white in urn A ($g$ is the draw of a black ball). Under ambiguity, the decision-maker tries to find the option that maximizes his or her utility of the worst outcome (Gilboa and Schmeidler 1989), which means minimizing the possibility of drawing a black ball from one of the urns in our example. This leads to an aversion to non-quantifiable probabilities and a preference for urn A.

Several studies refer to MEU (Kochov 2015; Maccheroni et al. 2006; Gilboa and Marinacci 2016), apply it (Bidder and Dew-Becker 2016; Zheng et al. 2015; Trojani and Vanini 2004), or extend it (Ghirardato et al. 2004; Li et al. 2016; Hansen and Sargent 2001; Chen and Epstein 2002; Miao and Wang 2011).

### Smooth ambiguity model

The two models described so far are based on a two-stage decision-making process. Before betting on an action, the decision-maker mentally estimates the probabilities of the actions. The Smooth Ambiguity Model (SAM) does not need the mental reduction of two-stage lotteries (Lang 2017).

Segal (1987, 1990) proposes two axioms, the *Reduction of Compound Lotteries Axiom* and the *Compound Independence Axiom,* which provide a new explanation for the Ellsberg paradox. Klibanoff et al. (2005) develop these further and designed the SAM. In this model, the decision criterion is based on a real-value function on a state space $S$. The decision-maker’s utility function $u$ follows von Neumann and Morgenstern (1944) and includes the risk attitude. The decision-maker’s attitude towards ambiguity is captured by $\phi$. $\mu$ is the subjective prior over $\Delta$, the set of possible probabilities over the state space, and $\pi$ a probability measure on the state space. The criterion results in

$$V\left( f \right) = \mathop \int \nolimits_{\Delta }^{{}} \phi \left( {\mathop \int \nolimits_{s}^{{}} u\left( f \right)d\pi )} \right)d\mu = E_{\mu } \phi \left( {E_{\pi } u \cdot f} \right).$$

A concave function of $\phi$ defines ambiguity aversion, while a convex function represents ambiguity seeking. When $\phi$ is linear, the decision-maker is ambiguity neutral and acts as an SEU maximizer. Assuming ambiguity aversion, Klibanoff et al. (2005) give the following example of the decision maker’s thinking:*”My best guess of the chance that the return distribution* [of an investment decision] *is ‘*$\pi$*’ is 20%. However, this is based on ‘softer’ information than knowing that the chance of a particular outcome in an objective lottery is 20%. Hence, I would like to behave with more caution with respect to the former risk.*”

After the introduction of SAM, many other papers followed the approach (Battigalli et al. 2015; Battigalli et al. 2016; Ju and Miao 2012; Maccheroni et al. 2013; Strzalecki 2013; Thimme and Völkert 2015; van de Kuilen and Wakker 2011; Wong 2015).

### Applications of the models

Models on ambiguity aversion have been used in various theoretical applications. Gao and Driouchi (2018), for instance, apply CEU to model the influence of ambiguity aversion on outsourcing decisions. Some papers study the role of ambiguity aversion in the design of auctions. Salo and Weber’s (1995) decision-makers, e.g., are characterized by CEU, Lo (1998) and Turocy (2008) use SEU with multiple priors as a framework, and the recent work by Koçyiğit et al. (2020) is based on MEU.

Analyzing investment decisions, Dow and Werlang (1992) use the CEU model for portfolio choices. Likewise, Berger et al. (2013) model the propensity of investments and portfolio compositions considering ambiguity aversion and learning. Escobar et al. (2015) investigate portfolio management for financial derivatives modeling ambiguity-averse investors. Using a similar approach, Bergen et al. (2018) study portfolios composed of derivatives and equities. Furthermore, Vardas and Xepapadeas (2015) analyze ambiguity aversion in portfolio selection using the Robust Portfolio Choice Theory.

Chateauneuf et al. (2000) apply the CEU model to the analysis of market equilibria and identify the decision-maker's tendency to diversify risk. Rigotti and Shannon (2005) use CEU to study factors influencing ambiguity aversion in the market with a model developed by Bewley (2002). Mukerji and Tallon (2001) attribute the imperfection of financial markets to ambiguity aversion utilizing the CEU model (see Rinaldi 2009 for a similar analysis with SAM). Chau and Vayanos (2008) analyze a market model in which some individuals have insider knowledge. Vitale (2018) extend this approach and apply it specifically to the insurance market. Also, Epstein and Wang (1994), Chen and Epstein (2002), and Liu (2011) study the impact of ambiguity aversion on market equilibria.

Epstein and Schneider (2008) model the valuation of assets for which decision-makers have access to information of different quality – assuming that low-quality information is associated with a high degree of uncertainty. Leippold et al. (2008) follow the same approach and extend the assumptions of Epstein and Schneider (2008) by learning of the decision-maker. The theoretical analysis of Dicks and Fulghieri (2019) implies that ambiguity aversion accelerated the financial crises. However, Condie (2008) argues that ambiguity aversion has very limited explanatory power for the long-term price development of assets.

## Evidence

In this section, we cluster the analyzed experimental and empirical literature into five categories discussing different forms of evidence on ambiguity aversion. The first subsection presents laboratory experiments testing the theoretical models from Sect. 2. The next subsection looks deeper into behavior in the domains of gains and losses, followed by evidence on ambiguity premiums. The last subsection reviews experimental and empirical applications on behavior under ambiguity.

### Testing the models

Halevy (2007) analyzes how far his experimental data can be explained by SEU, MEU, *Recursive Non-Expected Utility*, or *Recursive Expected Utility*. None of these models universally represents all the students' preferences. Similarly, Eichberger et al. (2012) do not find evidence for ambiguity-aversion when not only probabilities but also the stake sizes are ambiguous. However, Ahn et al. (2014) reveal that most of their subjects express SEU preferences and ambiguity aversion in line with MEU and CEU. Hey et al. (2010) are better able to explain behavior in their experiment with relatively simple models like the MEU compared to more sophisticated models like the CEU.

Stahl (2014) examines the preferences of decision-makers in a two-urn design. The subjects can decide to bet on a risky urn with a 50% probability to win $10 or on an ambiguous urn leading to a win of $10, $12, or $15. Most of the subjects prefer the risky over the $ 10 ambiguous urn. A large number of subjects are indifferent between the risky and the $12 ambiguous urn, and the majority prefer the $15 ambiguous over the risky urn. Stahl (2014) categorizes the subject pool into three groups: (1) subjects who behave according to SEU (12%), (2) subjects who behave according to MEU (26%), and (3) subjects whose decisions cannot be assigned to a theoretical model (60%). In line with his findings, most of the subjects in Charness et al. (2013) cannot be classified as ambiguity-averse.

### Gains versus losses

Curley and Yates (1989) let subjects choose between a risky bet (with a 25% probability of winning) and a draw from an urn representing ambiguity with 5 winning, 55 losing, and 40 unknown balls. In contrast to the Ellsberg paradox, most of the subjects prefer the ambiguous urn, either because of the rather low winning probability in the risky bet or because of optimism (see also Dimmock et al. 2013, 2016b; Kahn and Sarin 1988).

Cohen et al. (1987) compare decisions on potential gains with those on potential losses. Similar to Kahnemann and Tversky (1979), the authors observe that the majority of decision-makers are ambiguity-averse in the domain of gains but indifferent in the domain of losses. Likewise, Friedl et al. (2014) assess the willingness to pay (WTP) for insurance policies and observe no differences in the preferences for risky or ambiguous options in the domain of losses. For comparable results with low winning probabilities (in the range from 0.1% to 30%) of the risky alternative in the domain of losses see Curley and Yates (1985), Einhorn and Hogarth (1986), Di Mauro and Maffioletti (1996), Lara Resende and Wu (2010) and Tymula et al. (2012). With moderate (30% to 60%) winning probabilities, subjects tend to decide in favor of the ambiguous option (Baillon et al. 2018b, 2018a; Chakravarty and Roy 2009; Ho et al. 2002; Liu and Onculer 2017). In line with the Ellsberg paradox, higher winning probabilities lead to ambiguity aversion (Abdellaoui et al. 2011).

### Ambiguity premium

The ambiguity premium is comparable to the risk premium and reflects the difference between the WTPs of the risky and ambiguous option. Grou and Tabak (2008) observe that business and economics students in Brazil (in contrast to students from the University of Chicago) do not want to pay a premium to reduce ambiguity. Trautmann and van de Kuilen (2015) review several studies on ambiguity premiums and suppose that the magnitude of the premiums depends on the valuation method, the stake size, and the incentive method. They leave a systematic analysis of the heterogeneity of ambiguity premiums to future research.

Analyzing the Standard & Poor’s (S&P) 500 index, Brenner and Izhakian (2018) find evidence for an ambiguity premium in the stock market. They assume that the equity premium in asset pricing theory contains both a risk and an ambiguity premium. Jeong et al. (2015) develop a method for separately measuring premiums for risks and ambiguity. They apply this method to the S&P 500 index and confirm that investors pay an ambiguity premium. Although there have been publications on ambiguity premiums, research on their causes and the factors that may increase or decrease these premiums is still pending.

### Experimental and empirical applications

Sarin and Weber (1993) compare the behavior of students with bank executives' choices in sealed-bid auctions and double oral auctions. They find evidence for ambiguity aversion in both samples. Koudstaal et al. (2016) observe that entrepreneurs express a similar level of ambiguity aversion compared to employees and managers. Furthermore, Sutter et al. (2013) show that even children between 10 and 18 years are ambiguity-averse.

In a lab in the field experiment in Ethiopia, Akay et al. (2012) find that farmers do not differ in their level of ambiguity aversion from Dutch students in a classroom experiment. Likewise, Engle-Warnick et al. (2007) investigate the decision-making behavior of Peruvian farmers about the use of new cultivation technologies. Combining survey data with a lab in the field experiment, they observe a positive correlation between ambiguity aversion and conservative choices concerning new technologies. Similarly, Ross et al. (2012) find that ambiguity-averse farmers from Laos have a lower propensity to use new rice varieties.

Dimmock et al. (2016a, 2016b) confirm ambiguity aversion for US and Dutch households using online experiments implemented in representative surveys. Dimmock et al. (2016b) conduct five experiments with subjects of the “RAND American Life Panel” on household portfolio choice puzzles. They show a negative association of ambiguity aversion with stock market participation, the fraction of financial assets, and foreign stock ownership. Wakker et al. (2007) experimentally analyze the WTP for insurances with a representative sample from the general Dutch public. Using in-depth individual interviews to gain more information about the decision-maker's choices, they find evidence for ambiguity seeking rather than aversion.

Muthukrishnan et al. (2009) analyze the tendency of customers to prefer well-known over unknown brands. They observe that ambiguity-averse subjects prefer established brands, even if the product specifications are inferior compared to the unknown brands. Liu and Colman (2009) examine the long-term change in preferences for marketing strategies with a repeated experiment. They find that the intensity of ambiguity aversion decreases with an increasing number of repetitions. The authors assess decisions on marketing strategies as a control for classical Ellsberg urn decisions.

Moreover, Berger et al. (2013) investigate patients' decisions regarding vaccination against swine flu. They find that medical advice intended to support patients' decisions does not consider their ambiguity aversion. Courbage and Peter (2021) extend this study by examining the influence of ambiguity on personal vaccination decisions. Hoy et al. (2014) show that patients are reluctant to use free genetic tests due to ambiguity aversion. Segal and Stein (2006) find evidence that ambiguity-averse defendants in court tend to prefer bench trials over jury trials whenever their acquittal chances are substantial. Otherwise, they prefer jury trials. Furthermore, Ryall and Sampson (2017) see that contract partners tend to assure themselves against ambiguous actions in joint contracts.

Analyzing data of mutual fund investors, Li et al. (2017) argue that investors seem to place greater weight on the worst signal when confronted with information of ambiguous quality. Breuer et al. (2016) analyze the level of capital reserves held by companies. They find that managers reduce capital reserves when their investors are more ambiguity-averse. Similarly, Antoniou et al. (2015) observe that an increase of ambiguity in the stock market yields outflows from equity funds. Finally, Anderson (2019) examines the behavior of investors during the financial crisis in 2008. She finds that market outcomes can change more abruptly under ambiguity than under risk.

## Interpretation and discussion of review results

The overview of the models in Sect. 3 shows that the theoretical literature on ambiguity aversion focuses on two main topics: decision theory and its application to economic behavior (see also Sect. 2.3 for the interpretation and discussion of the bibliometric results). Remarkably, the application-oriented publications frequently refer to financial economics. Not often, the models are tested for their robustness in other economic contexts. This may explain why some researchers in this field suggest focusing more on natural experiments (see Sect. 6.1).

We see that the discussed theoretical models are frequently criticized for not delivering a realistic picture of human decision processes. Research on ambiguity aversion started with the criticism of SEU. Even Savage violated “his” *Sure Thing Principle* in the experiment of Allais and Hagen (1979) and the Ellsberg experiment. However, in *Foundations of Statistics* (1954) he shows a representation of Allais' paradox with which he wants to affirm his axiom (similar to our Table 4). Tversky and Slovic (1974) conduct an experiment in which subjects are allowed to retract their decisions if they have violated the axiom. Yet most of them stick to their first intuitive decision. Epstein (2010), Baillon et al. (2012), and Halevy and Ozdenoren (2008) criticize SAM by Klibanoff et al. (2005) for distinguishing between different forms of ambiguity that are not perceived differently by decision-makers. Klibanoff et al. (2012) respond that Epstein's (2010) thought experiments rather support their model than MEU. Furthermore, Machina (2009, 2014), l’Haridon and Placido (2010), and Baillon et al. (2011) criticize CEU by Schmeidler (1989) for contradicting robust experimental evidence.

Ellsberg (2011) notes that large parts of the literature misinterpret his paper in 1961 and that ambiguity aversion may not be a robust phenomenon: „*…I repeatedly mentioned that some subjects deliberately and consistently chose the more ambiguous alternative, rather than choosing to ‚avoid ambiguity ‘*…*My long-term complaint is not about the mischaracterization of my own exposition but about the general failure to explore this phenomenon in subsequent experiments and analysis.*“ While we agree that ambiguity aversion should not be considered in isolation, we disagree on a general failure to explore this aversion experimentally. Section 4 presents robust evidence on ambiguity aversion and little evidence on ambiguity seeking. It should be investigated which factors lead decision-makers to behave more ambiguity-averse (or ambiguity-seeking).

In contrast to the criticism of the models described in Sect. 3, experimental results on ambiguity aversion are less often discussed. One exemption is the general criticism of the artificial environment of laboratory experiments, which again speaks for more field or natural experiments (Trautmann and van de Kuilen 2015).

## Future research

The bibliometric analysis of our paper can be used as a basis for further research. We aim to set a starting point for identifying research trends and potential for future research (Sects. 2.2 and 2.3). Appendices A–E show the keyword co-occurrence by period. It can be seen that the topic areas in research on ambiguity aversion diversified over the years. In the period 1961–2000, the keywords *ambiguity*, *probability theory*, and *decision making* are predominant. In the following decades, new fields of applications emerged, e.g., *financial markets* (in 2006–2010) and *assets (accounting)*, *investments*, as well as *portfolio management* (in 2011–2015). This development indicates a trend towards application-oriented research on ambiguity aversion, which is likely to continue.

Some of the papers reviewed in our study suggest a high potential for future research. For instance, Gilboa and Marinacci (2016) see the opportunity to apply the ambiguity research to Akerlof ‘s (1970) “Market for Lemons”. A general advice is to focus more on natural experiments (Camerer and Weber 1992; Ellsberg 2011; Heath and Tversky 1991; Trautmann and van de Kuilen 2015). Baillon et al. (2018a) present a method for the experimental evaluation of natural decision problems under ambiguity. They distinguish two indices for ambiguity attitudes: ambiguity aversion and ambiguity perception. Further applications and critical examinations of this method concerning its validity are still needed. Moreover, future research could investigate factors influencing ambiguity premiums. Several real-life problems are not yet addressed by this literature stream, such as medical or consumption decisions.

Al-Najjar and Weinstein (2009) question numerous developments in theoretical and experimental research on ambiguity aversion. They demand a clearer delineation of theoretical models from each other – especially concerning descriptive versus normative models. Descriptive models should describe experimentally verifiable decision-making processes, while normative models are based on the rationality hypothesis. Also, they call for a more critical examination of the entire research on ambiguity aversion. Al-Najjar and Weinstein (2009) argue that the empirical results are not only explained by the models presented in Sect. 3 but also by models on heuristics (Samuelson 2001).

Ellsberg (2011) himself calls for a reorientation of decision-theoretical research and a less narrow investigation of ambiguity aversion. He recommends an examination of ambiguity – regardless of whether the decision-maker prefers or rejects it. Based on our results, we still see the demand for testing different models on ambiguity aversion experimentally. In line with Ellsberg (2011), one can question how far ambiguity aversion is a real phenomenon. We did not identify a lot of applications of the Ellsberg experiment outside the laboratory. Since the global economic situation in 2020 and 2021 is characterized by great uncertainty concerning future market developments, especially due to the Covid 19 pandemic, new opportunities for natural experiments emerge.

Information technology research has paid special attention to the development of artificial intelligence. The characteristics of ambiguity aversion in such artificially created, intelligence-based systems could be examined concerning similarities and differences to previous findings. Peysakhovich and Naecker (2017) provide a first approach, searching for alternative models of machine learning that could evaluate models of decision-making under risk and ambiguity. Moreover, research on ambiguity aversion could benefit from agent-based simulations (Georgalos 2018). Another promising tool to analyze if people are indeed ambiguity-averse is functional magnetic resonance imaging (fMRI) from neuroeconomics (Camerer et al. 2007; Hall et al. 2021).

## Conclusion

The results of our bibliometric analysis indicate a research boom on ambiguity aversion, especially in the last two decades. This research is published in highly ranked journals. The co-occurrence analysis of keywords reveals a focus on two specific main categories: the modeling of the decision process affected by ambiguity and the analysis of the effects of ambiguity aversion on economic decision problems, such as portfolio choices or investment decisions. The trend of the number of publications suggests that many more insights on ambiguity aversion can be gained in the coming years.

Our literature review shows that the theoretical models on ambiguity can only partly be verified experimentally or empirically. One of the main reasons for this is the complexity of the models. Furthermore, the subjects’ thought processes are difficult to trace. However, we can expect new contributions from agent-based models, in which the impact of different preferences can be directly tested, or neuroeconomic approaches, which try to open the black box of decision making.

One of the experimental findings is that the probability of the risky alternative influences ambiguity attitudes in Ellsberg paradoxes – from ambiguity seeking with low probabilities to ambiguity aversion with moderate or high probabilities. The mixed evidence for different models on ambiguity aversion encouraged a discussion on the robustness of the phenomenon (Baillon et al. 2012; Epstein 2010; Klibanoff et al. 2012; Machina 2009). l’Haridon and Placido (2010), e.g., show that the “Machina paradoxes” of CEU also apply to other models like MEU or SAM.

Our review reveals that there is still a large potential for future research, for instance on the heterogeneity of preferences under ambiguity – in the lab and especially in the field. Thus, research designs should emancipate from the Ellsberg-urn design and develop new methods for exploring ambiguity aversion. Anderson’s (2019) study on the role of ambiguity during the financial crisis may serve as an example. The corona crisis provides further potential for studying natural experiments on behavior under ambiguity.

## Appendix

### A. Key-Word Co-Occurrence (1961-1999)

We set the minimum threshold for occurrence of the keywords at 5. 7 of the 157 keywords met this criterion.

### B. Key-Word Co-Occurrence (2000–2005).

We set the minimum threshold for occurrence of the keywords at 5. 7 of the 201 keywords met this criterion.

### C. Key-Word Co-Occurrence (2006–2010).

We set the minimum threshold for occurrence of the keywords at 5. 22 of the 530 keywords met this criterion.

### D. Key-Word Co-Occurrence (2011–2015).

We set the minimum threshold for occurrence of the keywords at 5. 71 of the 1167 keywords met this criterion. We had to remove 3 keywords manually.

### E. Key-Word Co-Occurrence (2016–2020).

We set the minimum threshold for occurrence of the keywords at 5. 55 of the 1291 keywords met this criterion. We had to remove 7 keywords manually.

### F. Citation numbers of theoretical models

**Table:**

Source	Google Scholar Citations	Web of Science Citations	Business Source Premier Citations
Gilboa and Schmeidler (1989)	5036	1854	707
Schmeidler (1989)	3776	3776	491
Klibanoff et al. (2005)	1860	687	286
Maccheroni et al. (2006)	1170	NA	195
Bewley (2002)	937	NA	NA
Segal (1987a)	447	183	63
Cerreia-Vioglio et al. (2011)	283	9	46
Ergin and Gul (2009)	214	NA	38
Siniscalchi (2009)	176	NA	35
Chateauneuf, Faro (2009)	151	64	27
Ahn (2008)	152	55	24
Gul and Pesendorer (2013)	1	30	0

Citation counts from Google Scholar and Business Source Premier were retrieved on May 3, 2021. Citation Counts from Web of Science were retrieved on October 2, 2021.

## Footnotes

1 Data retrieved from Google Scholar only serves as additional information in our study because of the database’s limitations concerning bibliometric research (Aguillo 2012; Harzing and Alakangas 2016).
